# Neutrophil-to-Lymphocyte Ratio and Platelet-to-Lymphocyte Ratio Predicts the Disease Activity in Patients with Paediatric Systemic Lupus Erythematosus: An Observational Cross-Sectional Study

**DOI:** 10.31138/mjr.040923.ntl

**Published:** 2024-01-31

**Authors:** Paromita Nath, Dibyendu Raychaudhuri, Himadri Sekhar Kisku, Biswabandhu Bankura, Kalpana Datta, Manab Nandy, Rakesh Mondal

**Affiliations:** 1 Department of Paediatric Medicine;; 2 Multidisciplinary Research Unit;; 3 Department of Pharmacology, Medical College Kolkata, India

**Keywords:** paediatric systemic lupus erythematosus, neutrophil lymphocyte ratio, platelet lymphocyte ratio, disease activity

## Abstract

**Background:**

To assess the association between Neutrophil-to-lymphocyte ratio (NLR) and Platelet-to-lymphocyte ratio (PLR) with a degree of activity of paediatric systemic lupus erythematosus (pSLE) in terms of Systemic Lupus Erythematosus Disease Activity Index (SLEDAI-2K) score.

**Methods:**

This observational cross-sectional study was conducted in Paediatric Rheumatology Clinic, Medical College Kolkata. Systemic lupus erythematosus was diagnosed in children based on the 2019 EULAR/ACR criteria and/or SLICC 2012 criteria. A total of 31 children were included in the study whereas patients with chronic illnesses were excluded.

**Results:**

The mean age of presentation was 6.87 years among 31 children with pSLE. The most common presenting feature is prolonged fever and pallor followed by renal involvement. Most of the children presented with active disease 24 (77.42%). The mean NLR and PLR ratios were 4.29 ± 2.45 (0.9 – 10.57) and 112.26 ± 45.08 (47.7 – 203.9) respectively. The average SLEDAI-2K score was 15.9 (± 7.33). The NLR ratio in children with active disease (SLEDAI-2K score > 6) was 4.68 ± 2.44 (1.54 – 10.57) and inactive disease (SLEDAI-2K score <6) was 2.94 ± 2.10 (0.9 – 5.7) with p 0.0002. The PLR ratio in children with the active and inactive disease was 125.91 ± 41.29 (54.2 – 203.9) and 65.45 ± 18.0 (47.7 – 91.2) respectively (p 0.008).

**Conclusion:**

The NLR and PLR ratio might be utilised as a trusted and cost-effective tool in the evaluation and prediction of pSLE disease activity.

## INTRODUCTION

Systemic lupus erythematosus (SLE) is one of the major chronic autoimmune inflammatory diseases presented with extensive clinical and serological manifestations. Three factors; autoreactive B cells, T cell dysregulation, and cytokine abnormalities were the main cause of the disease.^1^Across the Globe, the incidence rate of paediatric SLE (pSLE) ranges between 0.3 to 0.9 per 100,000 per year whereas the prevalence rate ranges from 0.3 to 8.8 per 100,000.^2^ The presentation, disease course, and outcomes of this disease are unpredictable. As compared to adults, paediatric lupus patients also showed severe disease course. A significant number of paediatric SLE patients exhibited high disease activity. Several studies reported that around 70% of SLE patients showed relapsing-remitting and active disease patterns. 3 Several differences have been established between pSLE and adult-onset SLE (aSLE) in terms of clinical manifestations and serological profiles.^4^ The pSLE exhibits more disease activity with a significant relation to anti-double-stranded (ds) DNA antibodies, in comparison to aSLE.^5^Systemic Lupus Erythematosus Disease Activity Index 2000 (SLEDAI-2K) was the commonest used scoring criteria to evaluate disease activity.^6^

Neutrophils, lymphocytes, and platelets play major roles in the course of pSLE. 7 Different studies aimed to establish markers of inflammation in autoimmune and inflammatory disorders. Recently studies revealed that Neutrophil lymphocyte ratio (NLR) platelet lymphocyte ratio (PLR), and, CBC parameters might be used as biomarkers for evaluating the disease activity.^8^ However, data are scarce and also as per our knowledge, there is no report from IndianpSLE patients regarding the NLR and PLR value. From this point of view, this study aimed to analyse the potential association between NLR, PLR, and disease activity in Indian pSLE patients.

## MATERIALS AND METHODS

### Study subjects

This is an observational study with a cross-sectional design conducted in Paediatric Rheumatology Clinic, Medical College Kolkata with purposive sampling. A total of 31 children aged less than 12 years diagnosed with systemic lupus erythematosus as per Systemic Lupus International Collaborating Clinics 2019 criteria were enrolled from the Paediatric Medicine department (both in-patient and outpatient) in the study. Patients with chronic illnesses like diabetes, acute viral illness, and other auto-immune diseases were excluded from the study. Ethical clearance from Institutional Ethical Committee (Ethical Ref No.: MC/KOL/IEC/NON-SPON/1812/03/2023, Dated-10/03/202) and written consent from parents/caregivers/legal guardians was taken before the conduction of the study. All essential data from history, clinical examination, and severity of disease activity were recorded in predesigned clinical proforma. The severity of the disease activity of pSLE was graded according to SLEDAI-2K. Based on the score, patients were categorised into two groups: (i) active disease: patients with SLEDAI-2K score ≥ 6, and (ii) inactive disease: patients with SLEDAI-2K score < 6.^9^

### Clinical Parameters

The laboratory investigations included: complete blood count (haemoglobin, total leucocyte count with differential count, platelet count), Antinuclear Antibody (ANA), anti-ds DNA, serum C3, serum C4, urine routine, and microscopy. Other investigations for the management of the patient were done by the treating unit as appropriate. The calculation of NLR and PLR values was done through a complete blood count.

### Statistical Analysis

A t-test was used for comparison between these two groups, while the Mann-Whitney test was used for non-normally distributed continuous variables. To compare two continuous variables Pearson’s correlation coefficient was used. A p-value of less than 0.05 was considered statistically significant. All the tests were done using the SPSS version 22.

## RESULTS

In this study, a total of 31 patients with pSLE were studied and the demographic characteristics of the studied subjects are presented in **Table 1**. The recruited patients were aged between 4–11 years with a mean age of 6.87 years. Most of the individuals were female (90.32%) as several authors reported that, the strongest risk factor for developing SLE is being female.^10^ The most common clinical features of our study participants were prolonged fever (70.97%), and pallor (70.97%) followed by renal (61.29%) and cutaneous (61.29%) involvement, while the least common symptoms were neurological disorders (9.68%), arthritis (19.35%), and serositis (22.58%) (**Table 1**). Our result is in line with a recent publication where authors revealed that renal disorders, malar rash, and oral ulcers were the most common clinical features while alopecia, headaches, and serositis were the least common symptoms.^9^

**Table 1.** Demographic characteristics of pSLE patients.

**Parameter**	**pSLE patients (n = 31) Mean ± SD (min–max) Median (IQR) or n (%)**

**Age (years)**	6.87 ± 2.32 (4 – 11)
	6 (5 - 10)

**Gender**	
Boy	3 (9.68)
Girl	28 (90.32)

**Malar rash/Cutaneous involvement**	
Yes	19 (61.29)
No	12 (38.71)

**Oral/Nasal ulcers**	
Yes	9 (29.03)
No	22 (70.97)

**Pallor**	
Yes	22 (70.97)
No	9 (29.03)

**Non-scarring alopecia**	
Yes	10 (32.26)
No	21 (67.74)

**Arthritis**	
Yes	6 (19.35)
No	25 (80.65)

**Serositis**	
Yes	7 (22.58)
No	24 (77.42)

**Prolonged fever**	
Yes	22 (70.97)
No	9 (29.03)

**Photosensitivity**	
Yes	13 (41.94)
No	18 (58.06)

**Seizure**	
Yes	3 (9.68)
No	28 (90.32)

**Headache**	
Yes	3 (9.68)
No	28 (90.32)

**Blurring of vision**	
Yes	0 (0)
No	31 (100)

The clinical characteristics of the patients are represented in **Table 2**. We divided the patient population into 2 groups based on disease activity (active- ≥6, inactive-<6) as per SLEDAI-2K score. A majority (77%) of patients were active and the remaining patients were inactive (**Table 2**). Autoimmune profile including ANA by IFA was positive in all individuals, while Anti-dsDNA was positive in 78.3% of patients, a result in line with several studies conducted in Pakistan and Southeast Asia.^9,11,12^ The mean of neutrophils was 7292.5 ± 3739.7 cells/mm^3^, the mean of lymphocytes was 2242.8 ± 1800.8 cells/mm^3^, the mean of platelets was 212806.5 ± 143113.3 cells/mm^3^ (**Table 2**). The finding is consistent with the Fikri et al., study, where the authors revealed that the mean of neutrophils, lymphocytes, and platelets was 5208.72 ± 2454.11 cells/mm^3^, 1378.82 ± 788.21 cells/mm^3^ and, 260341 ± 111409 cells/mm^3^ respectively.^13^

**Table 2.** Laboratory findings in pSLE patients.

**Parameter**	**pSLE patients (n = 31)**
	**Mean ± SD (minimum – maximum)**
	**Median (IQR) or n (%)**

**Haemoglobin (mg/dl)**	8.8 ± 2.5 (3.5 – 12.9)
	9.1 (7.9 – 10.6)

**Total leucocyte count**	10213.55 ± 4940.5 (3380 – 21500)
	9900 (6000 - 142000)

**Neutrophil count**	7292.5 ± 3739.7 (2366 - 13960)
	6160 (4500 - 11700)

**Lymphocyte count**	2242.8 ± 1800.8 (709 - 7175)
	1461 (1120 - 2570)

**Platelet**	212806.5 ± 143113.3 (86000 – 612000)
	166000 (138000 - 250000)

**Neutrophil to lymphocyte ratio**	4.29 ± 2.45 (0.9 – 10.57)
	3.8 (2.77 – 5.7)

**Platelet to lymphocyte ratio**	112.26 ± 45.08 (47.7 – 203.9)
	99.8 (85 – 143.7)

**Proteinuria**	
Yes	19 (61.29)
No	12 (38.71)

**Haematuria**	
Yes	17 (54.83)
No	14 (45.17)

**Pyuria**	
Yes	16 (51.61)
No	15 (48.39)

**Urinary cast**	
Yes	18 (58.06)
No	13 (41.94)

**Antinuclear Antibody**	
1:160	24 (77.42)
1:320	7 (22.58)
	
**anti dsDNA (IU/ml)**	154.9 ± 198.9 (10 - 680)
	85 (67.4 – 93.5)

**Serum C3 (mg/dl)**	52.64 ± 18.66 (26.6 – 90.3)
	46.8 (39 - 66)

**Serum C4 (mg/dl)**	5.27 ± 1.98 (1.1 – 8.42)
	5.28 (3.6 – 7.29)

**SLEDAI-2K score**	15.9 ± 7.33 (5 – 33)
	15 (11 - 20)

**Disease severity**	
active [≥6]	24 (77.42)
inactive [<6]	7 (22.58)
	
**Hospital stay (days)**	36.4 ± 16.7 (12 - 59)
	35 (19 - 52)

**Need for PICU stay, n (%)**	
Yes	13 (41.94)
No	18 (58.06)

Final disposition	
Discharge	29 (93.55)
Death	2 (6.45)

**Figure 1.**
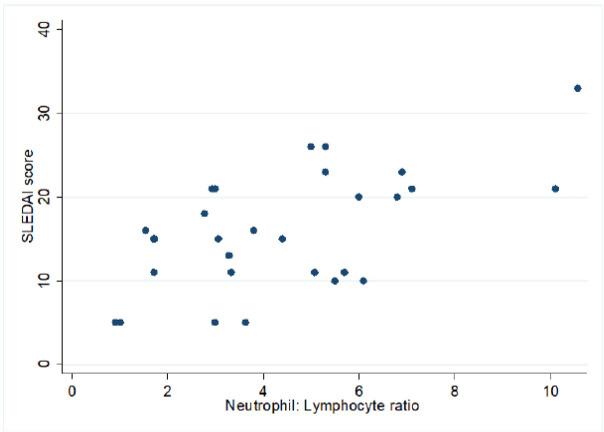
Correlation of NLR scores with SLEDAI-2K scores.

**Figure 2.**
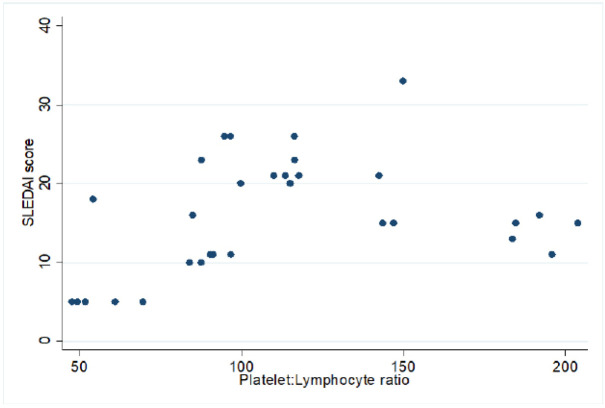
Correlation of PLR scores with SLEDAI-2K scores.

We evaluate the association between NLR and PLR with the degree of disease activity (**Table 3**). Our result exhibited NLR and PLR both are significantly associated with disease activity (**Table 3**). The median ratio of NLR and PLR is significantly increased in active patients in comparison to inactive patients. Our result also showed that the NLR had a significant positive correlation with the SLEDAI-2K scoring (**Figure 1**, r=0.6, p-value <0.001). In addition, the PLR had a positive correlation with the SLEDAI-2K scoring (**Figure 2**, r=0.31, and p-value <0.001).

**Table 3.** Disease activity in D2T axSpA and the non-D2T axSpA groups.

**Parameter (Mean ± SD)**	**Degree of activity of pSLE**		**P value**
	**Active (n-24)**	**Inactive (n-7)**	
**Neutrophil to lymphocyte ratio**	4.68 ± 2.44 (1.54 – 10.57)	2.94 ± 2.10 (0.9 – 5.7)	0.0002
**Platelet to lymphocyte ratio**	125.91 ± 41.29 (54.2 – 203.9)	65.45 ± 18.0 (47.7 – 91.2)	0.008

## DISCUSSION

Current knowledge on the role of NLR and PLR in disease (pSLE) prediction is limited. Our study first aimed to establish the role of NLR and PLR in SLE evaluation and prediction in paediatric patients from India. The study results revealed that PLR and NLR were statistically high in active pSLE patients in comparison to inactive patients.

The majority of the patients in our study had active disease (SLEDAI-2K score > 6), namely, 24 patients (77.42%), and the rest 7 children (22.58%) had inactive disease. The finding is compatible with a study by Shamim et al. which showed that the SLEDAI-2K score was ≥6 in 87% (n=20) while less than 6 in 13% (n=3) of patients.^10^

Our results found that the average NLR ratio is 4.29 (± 2.45), the average PLR ratio is 112.26 (± 45.08), and the average SLEDAI-2K score is 15.9 (± 7.33). This finding is compatible with a study by Qin et al. which showed the mean NLR and PLR ratio to be 3.61±2.04 and155.64±91.69 respectively.^14^

Our study also suggests a statistically significant (p = 0.0002) difference between NLR ratio in children with active [4.68 ± 2.44 (1.54 – 10.57)] and inactive [2.94 ± 2.10 (0.9 – 5.7)] disease as per SLEDAI-2K score. In addition, NLR had a positive correlation with the SLEDAI-2K, which is consistent with the study by Fikri et al.^13^ They exhibited a significant relationship between the NLR ratio and the degree of disease activity of pSLE. Additionally, a case-control study conducted by Oehadian et al, showed that NLR was significantly higher in SLE patients.^15^ Another study by Qin et al. showed a positive correlation of NLR with SLEDAI-2K (r=0.471, p<0.01).^14^

The PLR ratio was significantly (p = 0.008) associated with the degree of disease activity of pSLE with a mean PLR ratio of 125.91 ± 41.29 (54.2 – 203.9) and 65.45 ± 18.0 (47.7 – 91.2) in active and inactive disease respectively in our study. In addition, our results also found that the PLR had a moderate correlation with the SLEDAI-2K score. Several studies supported our finding as PLR was positively correlated with systemic lupus erythematosus disease activity.^13,14,16^ Our study has some limits. Our study was conducted in a single centre; hence, our sample size is small. In addition, the effect of treatment on NLR or PLR was not explored due to inadequate data and only SLEDAI-2K for scoring.

## CONCLUSION

This study concluded that NLR and PLR were significantly increased in SLE patients and were positively correlated with disease activity. These findings of the study indicate that NLR and PLR might be used as potential tools for the determination of inflammation and assessing the activeness of the disease.
